# A Therapeutic Sheep in Metastatic Wolf’s Clothing: Trojan Horse Approach for Cancer Brain Metastases Treatment

**DOI:** 10.1007/s40820-022-00861-1

**Published:** 2022-04-28

**Authors:** Hai-jun Liu, Mingming Wang, Shanshan Shi, Xiangxiang Hu, Peisheng Xu

**Affiliations:** Department of Drug Discovery and Biomedical Sciences, College of Pharmacy, University of South Carolina, Columbia, SC 29208 USA

**Keywords:** Trojan horse, Brain metastasis, Blood–brain barrier, Cell membrane, Nanomedicine

## Abstract

Early-stage brain metastasis of breast cancer (BMBC), due to the existence of an intact blood–brain barrier (BBB), is one of the deadliest neurologic complications. To improve the efficacy of chemotherapy for BMBC, a Trojan horse strategy-based nanocarrier has been developed by integrating the cell membrane of a brain-homing cancer cell and a polymeric drug depot. With the camouflage of a MDA-MB-231/Br cell membrane, doxorubicin-loaded poly (D, L-lactic-co-glycolic acid) nanoparticle (DOX-PLGA@CM) shows enhanced cellular uptake and boosted killing potency for MDA-MB-231/Br cells. Furthermore, DOX-PLGA@CM is equipped with naturally selected molecules for BBB penetration, as evidenced by its boosted capacity in entering the brain of both healthy and early-stage BMBC mouse models. Consequently, DOX-PLGA@CM effectively reaches the metastatic tumor lesions in the brain, slows down cancer progression, reduces tumor burden, and extends the survival time for the BMBC animal. Furthermore, the simplicity and easy scale-up of the design opens a new window for the treatment of BMBC and other brain metastatic cancers.

**Supplementary Information:**

The online version contains supplementary material available at 10.1007/s40820-022-00861-1.

## Highlights

- DOX-PLGA@CM employs the whole set of membrane molecules of a brain-homing metastatic breast cancer cell optimized through a natural selection process. Thus, the hetero and multivalent effects of these molecules greatly facilitate the nanoparticle crossing the blood-brain barrier.
- Attributed to the homotypic effect of the nanocarrier, DOX-PLGA@CM shows stronger anticancer efficacy than free DOX for its parenteral cells.
- DOX-PLGA@CM effectively reaches the metastatic tumor lesions in the brain, and slows down the progression of brain metastatic breast cancer.

**Supplementary Information:**

The online version contains supplementary material available at 10.1007/s40820-022-00861-1.

## Graphical abstract

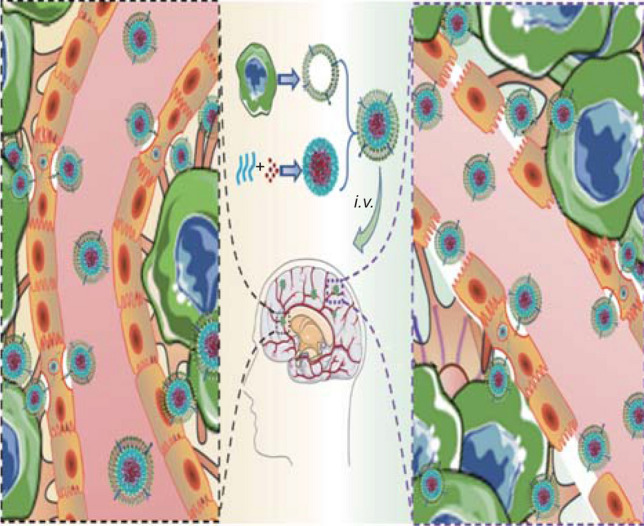

## Introduction

Brain metastases of breast cancer (BMBC) are one of the most frequent and deadliest neurologic complications [1]. More than one-third of Her2 positive or “triple-negative" (estrogen receptor‐negative, progesterone receptor‐negative, and Her2‐negative) breast cancer patients will progress to brain metastasis, which has a poor prognosis with a median survival time of fewer than 12 months [2, 3]. Typically, the unrestrained brain metastasis presents the feature of aggressive infiltration, leading to the destruction and displacement of brain tissue and subsequently cognitive impairment [4]. Multifocal lesion distribution through the whole brain is another significant feature of brain metastasis at the time of diagnosis, as evidenced by the localization of the lesions in the cerebral hemispheres, cerebellum, and brainstem are 80%, 15%, and 5%, respectively [5].

Currently, BMBC treatment mainly relies on surgery and radiation, including stereotactic radiosurgery (SRS) and whole-brain radiation therapy (WBRT) [6]. Surgery is only limited to some well-defined and non-invasive metastasis lesions in favorable locations. Radiation therapy, especially WBRT, is challenged by significant side effects such as cognitive impairment [7, 8], while the improved overall survival time is limited and heterogeneous [9]. However, chemotherapy, a widely adopted therapeutic approach for many cancers, is excluded from the standard care for BMBC due to its inability of transporting to brain metastases to reach adequate therapeutic concertation in the presence of blood–brain barrier (BBB) and/or blood–tumor barrier (BTB) [1]. At the early stage, BMBC displays a co-opted proliferation and growth pattern along the BBB basement membrane, and the BBB integrity is relatively well maintained. Along with cancer progression, neovascularization would sprout out from existing metastatic lesions with the physiologically compromised structure of BBB, termed as BTB [10]. Although the emergence of BTB at the advanced stage of BMBC allows for some extravasation of larger molecules, including nanoparticles, it is still not sufficient for the accumulation of drugs and nanoparticles to a therapeutically effective concentration [11, 12]. Moreover, the permeability of BTB is of significant heterogeneity in different metastatic lesions, even within a lesion [12]. Unfortunately, the progression from BBB to BTB indicates the further deterioration and poor prognosis of BMBC. Therefore, there is an urgent need to develop a reliable and practical approach to treat BMBC at its early stage when the BTB has not yet emerged.

To help drugs cross the BBB, many nanoparticle-based delivery systems have been developed due to their improved circulation time in the blood and BBB-oriented functionalization potential. For instance, by conjugating ligands for the receptors, such as lactoferrin [13] and transferrin [14], express on the surface of brain endothelial cells on the nanoparticles, an improved cargoes delivery to the brain could be realized by receptor-mediated transcytosis [15]. Nonetheless, due to the competitive binding with the abundant endogenous ligands [16], reduced targeting property induced by the rapid formation of plasma protein corona on the nanoparticle surface in circulation system [17], and difficulty in bottom-up targeting ligand conjugation [18], the accumulation of nanoparticles in the brain via receptor-mediated transcytosis is still too low to elicit an effective therapeutic responsive [19].

Recently, emerging biomimetic nanotechnology realized through cell membrane camouflage has attracted tremendous attention [18, 20]. In this notion, a synthetic nanoparticle is cloaked with a natural cell membrane to yield a core/shell structure and bestow the nanoparticle with naturally evolved properties of the source cells, such as immune escaping capacity and prolonged blood circulation time [18]. Encouraged by these advantages, researchers have adopted this strategy for the treatment of brain-related diseases, including glioblastoma multiforme [21, 22], ischemic stock [23, 24], and Parkinson’s disease [25]. Still, very few explored that in cancer brain metastases.

**Scheme 1:**
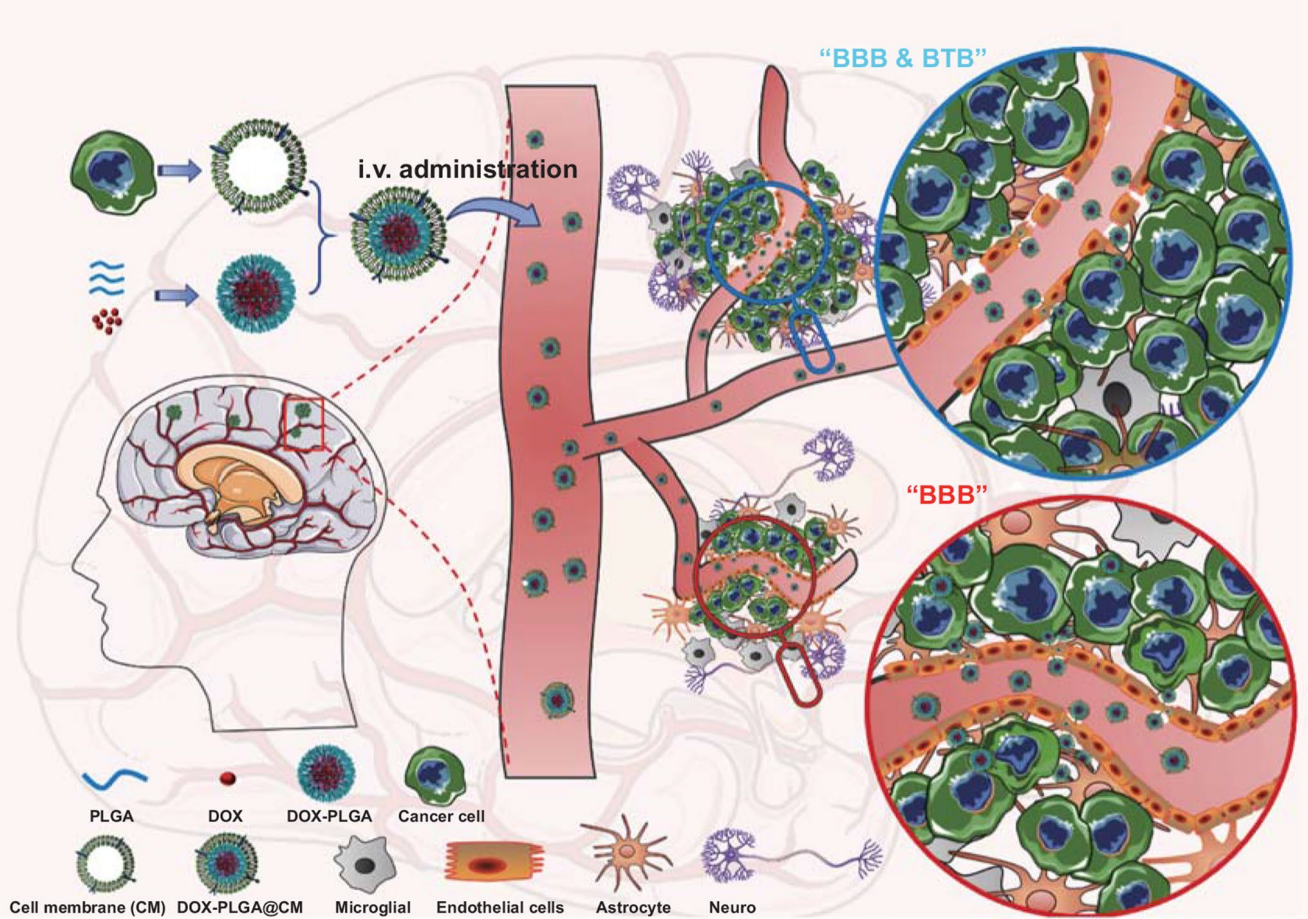
The schematic for the fabrication of DOX@PLGA@CM and its in vivo application for the treatment of brain metastases of breast cancer

During the cascade of brain metastases formation [26], disseminated cancer cells from the primary tumor site arrive in brain vasculature as the “seeds.” Subsequently, they traverse across the BBB into the brain parenchyma. It is believed that the interactions between the membrane molecules and receptors of the cancer cells and the endothelial cell are critical for the attachment of cancer cells to brain endothelial cells and subsequent trans-BBB migration [1, 26]. Since the above cell–cell interaction is multivalent and involves many substances [26], the BBB penetrating efficacy of brain metastatic cells is superior to most developed brain targeted systems. Inspired by this process, we developed a Trojan horse strategy by integrating a polymeric nanoparticle and the cell membrane from a brain homing breast cancer cell, which was generated after two rounds of intracardiac injection and resection from the brain. During this process, the expressed membrane molecules of the cancer cells have been optimized in vivo, which would endow the nanocarrier with the capability of crossing the BBB, homing to brain metastasis lesion, and realizing effective chemotherapy for BMBC (Scheme 1). The biomimetic nanocarrier has a core–shell nanostructure, where doxorubicin (DOX) loaded polymeric nanoparticle prepared from poly (D, L-lactic-co-glycolic acid) (PLGA) constitutes the core, and cell membrane (CM) derived from brain homing MDA-MB-231 breast cancer cell (MDA-MB-231/Br) conceals the core to yield a DOX-PLGA@CM. To best recapitulate the condition of the early-stage of BMBC, a brain metastasis cancer model constructed through systemic inoculation (intracardiac injection) of MDA-MB-231/Br cells, which maintains the integrity of BBB contrasting to the widely adopted local intracranial injection [27], was employed for the biodistribution and in vivo therapeutic efficacy study.

## Materials and Methods

### Materials

Poly(D, L-lactide-co-glycolide) (PLGA, acid terminated, lactide/glycolide 50:50, Mw 38,000–54,000 Da), Poly(vinyl alcohol) (PVA, Mw 9000–10,000) and Nile Red were purchased from Sigma-Aldrich Chemical Co. (St. Louis, MO, USA). BCA Protein Assay Kit (BCA), 1,1'-Dioctadecyl-3,3,3',3'-Tetramethylindotricarbocyanine Iodide (DIR), Hoechst 33,342, Gibco Dulbecco's modified Eagle medium (DMEM), fetal bovine serum (FBS), trypsin–EDTA free (0.25%), penicillin–streptomycin (PS) were purchased from Thermo Fisher Scientific, Inc. (Waltham, MA, USA). Doxorubicin hydrochloride (DOX) was purchased from LC Laboratories (Woburn, MA, USA). (3-(4,5-dimethylthiazol-2-yl)-2,5-diphenyltetrazolium bromide (MTT) was purchased from Tokyo Chemical Industry Co., Ltd (Portland, OR, USA). EDTA-free mini protease inhibitor tablet was supplied by Roche Applied Science (Indianapolis, IN, USA). D-luciferin potassium salt was purchased from PerkinElmer, Inc. (Waltham, MA, USA). All other chemical reagents and solvents used in this research were purchased from Sigma-Aldrich and used directly without further process unless specially noted.

### Fabrication of DOX-PLGA

The emulsion solvent evaporation method was used to prepare DOX-loaded PLGA nanoparticles (DOX-PLGA). In brief, 100 mg of PLGA and 5 mg of DOX was dissolved in 2 mL of chloroform, followed by the addition of 50 µL of triethylamine and sonicating for 1 min using a sonicator (ULTRASONIC PROCESSOR XL, Misonix, NY, USA) at 70% pulse duty cycle on ice. The solution was dropwise added into 10 mL of 5% PVA solution under slight vortex and further sonicated under the same output and frequency for 15 min on ice. The emulsion solution was slowly poured into 20 mL of 0.5% PVA solution under stirring and continued to stir for 12 h under atmospheric pressure at room temperature overnight until the total evaporation of the organic solvent. The DOX-PLGA were harvested by centrifugation (13,500 g for 15 min), washed twice with PBS solution, and stored at 4 °C for further use.

### Cell Culture

Human breast cancer cells MDA-MB-231 were purchased from ATCC, and its subtype of MDA-MB-231/Br cells, a brain-homing derivative of a human breast adenocarcinoma line MDA-MB-231, were purchased from Memorial Sloan Kettering Cancer Center. The cells were cultured in Dulbecco's Modified Eagle's Medium (DMEM) containing 10% of fetal bovine serum (FBS, Gibco), 100 U mL^−1^ of penicillin and 100 mg mL^−1^ of streptomycin under a humidified atmosphere of 5% CO_2_ at 37 °C. The culture medium was replaced with a fresh one every two days.

### Fabrication of DOX-PLGA@CM

Firstly, the cell membrane vesicles (CM) of MDA-MB-231/Br were prepared as our previous method [28]. MDA-MB-231/Br cells were a kind gift from Dr. Joan Massagué at the Memorial Sloan Kettering Cancer Center. Luciferases stably expressing cells were established by lentivirus transfection of Luc vector. Briefly, MDA-MB-231/Br cells were harvested with 2 mM EDTA PBS solution and resuspended in hypotonic lysis buffer (20 mM Tris–HCl pH7.4, 10 mM KCl, 2 mM MgCl_2_, and 1 mM EDTA-free mini protease inhibitor tablet per 10 ml), followed by homogenization with a Dounce homogenizer for 20 times. The homogenate was centrifuged at 10,000 g for 10 min, and the supernatant was collected and further ultracentrifuged at 100,000 g for 1 h. The cells pellet was collected and washed once with 10 mM Tris–HCl (pH = 7.5). Finally, the cell ghosts were re-suspended in water, sonicated for 30 s in a water bath sonicator, followed by physical extrusion through a 400 nm polycarbonate membrane for 5 circles to obtain CM vesicles. The protein concentration of the CM vesicles was quantified by BCA protein assay. The CM vesicles were stored at 4 °C until further use. DOX-PLGA and CM vesicles were sufficiently mixed at 1:1 weight ratio of PLGA to protein and further extruded through a 200 nm polycarbonate membrane for 7 circles to fabricate CM coated DOX-PLGA (DOX-PLGA@CM).

### Characterization of the Nanoparticles

The morphologies of DOX-PLGA and DOX-PLGA@CM were characterized by transmission electron microscope (Hitachi HT7800 TEM, Hitachi High Technologies, Tokyo, Japan), and their hydrodynamic sizes and zeta potentials were measured by Nano ZS Zetasizer (Malvern Instruments, UK). DOX loading content (LC) and loading efficiency (LE) were measured by using UV–Vis spectroscopy at the wavelength of 480 nm or fluorescence spectroscopy at excitation/emission wavelength of 480/570 nm with free DOX as a standard after their liberating from nanoparticles by DMSO according to the following equations, LC = (amount of loaded DOX)/(amount of loaded DOX + amount of PLGA) × 100 and LE = (amount of loaded DOX)/(amount of total DOX) × 100. The cell membrane proteins coated on the surface of nanoparticles were confirmed and characterized by sodium dodecyl sulfate polyacrylamide gel electrophoresis (SDS-PAGE) and stained with Coomassie brilliant blue (Invitrogen, Oregon, USA) method. To characterize the drug release profiles of DOX, DOX-PLGA@CM dispersed in pH 7.4 or pH 5.0 PBS were placed in a dialysis bag with 8000 MWCO and immersed in their corresponding PBS buffers. At given time intervals, 5 mL dialysates were gathered to quantify DOX release, and the same volume of fresh buffer was replenished.

### Cellular Uptake of the Nanoparticles in Cancer Cells

To investigate the cellular internalization of the nanoparticles, MDA-MB-231 and MDA-MB-231/Br were cultured in 35 mm glass-bottom dishes at a density of 20,000 per well. After 24 h of culture, the medium was replaced with a fresh one containing DOX, DOX-PLGA and DOX-PLGA@CM at a DOX concentration of 0.5 µM. After 3 h of incubation, the cells were washed with cold PBS and fixed with 4% paraformaldehyde for 10 min. The nuclei were stained with 10 μg mL^−1^ of Hoechst 33,254 for 8 min at room time. The fluorescence images were observed with Carl Zeiss LSM700 confocal microscope, where red signal indicated the uptake of DOX and green signal (GFP) outlined the cell. Flow cytometry (FCM) was further used to quantitatively measure the internalization of nanoparticles into NIH3T3, MDA-MB-231 and MDA-MB-231/Br cells with a flow cytometer (BD Accuri C6, BD Biosciences) at *λ*_ex_ 488 and *λ*_em_ 560 nm. The signals of three samples for each treatment were collected till reaching 20,000 events. Forward and side-scatter were “gated” to exclude dirt and clumped cells. Identical laser setting and gating were used for the analyses of all samples.

### In Vitro* Cytotoxicity Assay*

The cytotoxicity of different nanoparticles to NIH3T3, MDA-MB-231 and MDA-MB-231/Br cells was investigated by MTT assay. In brief, cells were seeded in 96-wells plate at a density of 8,000 cells per well and cultured for 24 h. Thereafter, the medium was replaced with fresh one containing different drugs in a serial of concentrations and incubated for another 48 h. After that, 10 μL of MTT solution (5 mg mL^−1^ in PBS) was added to each well and incubated for another 4 h. Then, the medium was discarded and replaced with 100 µL DMSO. The optical density (OD) of each well was measured at 570 nm, and untreated cells were used as controls. The cell viability was calculated as the following equation: OD_A_/OD_B_ × 100%, where OD_A_ is the OD value of experimental group cells, and the OD_B_ is the OD value of control cells.

### Cellular Uptake of Nanoparticles in hCMEC/D3 Cells

Nile red was loaded into PLGA nanoparticles (Nile-PLGA) to track nanoparticles intracellular distribution. Nile-PLGA was fabricated as DOX-PLGA, excepting the substitution of DOX with Nile red. hCMEC/D3 were cultured in 35 mm glass-bottom dishes at a density 2 × 10^4^ cells/well. After 24 h of culture, the cells were treated with Nile-PLGA and Nile-PLGA@CM at a Nile red concentration of 0.1 µg mL^−1^ and incubated for 2, 4, and 6 h. Then, the cells were washed three times with cold PBS, and fixed with 4% paraformaldehyde for 10 min. Hoechst 33,254 was used to stain the nuclei of the cells. The uptake of nanoparticles in hCMEC/D3 was characterized by the Carl Zeiss LSM700 confocal microscope.

### In Vitro* BBB Penetration Assay*

The BBB model was constructed following a reported method [29]. In brief, 20,000 hCMEC/D3 cells were seed on a polycarbonate 24-well Transwell membrane with 8 μm mean pore size to form a cell monolayer. The trans-endothelial electrical resistance (TEER) of the hCMEC/D3 cell monolayer was measured every day by an epithelial voltohmmeter (Millicell-RES, Millipore, USA). Until the TEER was above 200Ω cm^2^, the established BBB model was used to estimate the penetrating ability and efficiency of the nanoparticles. Culture media (200 µL) containing Nile-PLGA or Nile-PLGA@CM were added into the upper chamber. The low chamber was filled with 600 µL of the plain medium. At given time intervals, the medium in the lower chamber was collected to quantify the penetrating efficiency of nanoparticles by fluorometry and replaced with a fresh medium. The penetrating efficiency of the Transwell without cell monolayer was used as a positive control [30].

### Breast Cancer Brain Metastases Model

All animal experiments were carried out following the protocol approved by the Institutional Animal Care and Use Committee (IACUC) of the University of South Carolina. Female BALB/c nude mice (5–6 weeks old) and C57 BL/6 J (5–6 weeks old) were purchased from Jackson laboratory. For tracking the tumor growth, MDA-MB-231/Br cells were transduced with pLentipuro3/TO/V5-GW/EGFP-Firefly Luciferase with the help of a lentivirus to yield a luciferase stably expressing cell line (MDA-MB-231/Br-Luc). Breast cancer brain metastases model was established following the method described in the literature [30, 31]. In brief, the BALB/c nude mice were anesthetized by 2% isoflurane, and 200,000 MDA-MB-231/Br-Luc cells in 100 µL DMEM medium were intracardiac injected into the left ventricle with a 26 G hypodermic needle. The behaviors of mice were observed every other day. The tumor growth was monitored by bioluminescence imaging using IVIS Lumina III whole-body imaging system (PerkinElmer Inc., Waltham, USA) twice per week.

### In Vivo Distribution of Nanoparticles

For tracking nanoparticles in vivo distribution in mice, DIR was loaded in nanoparticles as a fluorescence probe. DIR-PLGA and DIR-PLGA@CM were injected intravenously to normal mice (C57 BL/6 J) or breast cancer brain metastatic BALB/c mice at a DIR dose of 0.5 mg kg^−1^. Three hours post-injection, the mice were anesthetized and imaged using an IVIS Lumina III imaging system (excitation: 750 nm; emission: 770–790 nm). After that, the mice were sacrificed, and the major organs, including brain, heart, liver, spleen, lung, and kidney were collected for ex vivo imaging to investigate nanoparticles tissue distribution.

### Blood Clearance Kinetics

C57 BL/6 J mice were divided into three groups and intravenously injected via tail vein with free DOX, DOX-PLGA, and DOX-PLGA@CM at a DOX equivalent dose of 2.5 mg kg^−1^. At predesigned time points (0.5, 1, 2, 4, 10, and 24 h), blood samples were collected from the orbital vein of the mice (*n* = 3). The DOX amount in the blood was quantitatively determined by a fluorospectrometer following our previously reported method [32].

### Antitumor Therapy in the Breast Cancer Brain Metastases Model

Once obvious brain metastatic tumor signal observed by the in vivo imaging system, around three weeks after the inoculation of the cancer cells, the mice were randomly divided into 4 groups, including saline, free DOX, DOX-PLGA, and DOX-PLGA@CM, and received intravenous administration of the corresponding treatments at a DOX equivalent dosage of 2.5 mg kg^−1^ twice per week. The progression of brain metastases was monitored by bioluminescence imaging twice per week.

### Histological Analysis

In a separate study, on day 15 (2 days after mice received the last treatment), three mice from each group were sacrificed, the brains were isolated for H & E staining to evaluate the antitumor effect, and other major organs including heart, liver, spleen, lung, and kidney were collected for H&E histological assay for toxicity evaluation.

### Statistical Analysis

All data were displayed as mean ± standard deviation (SD) (*n* ≥ 3), and the statistical significance was analyzed by GraphPad Prism 7.0 (GraphPad Prism Software Inc., San Diego, California) using Student's t test or ANOVA with Tukey’s significant, excepting Mantel Cox-test for survival analyses. Differences were considered significant when the p value was less than 0.05.

## Results and Discussion

### Nanoparticle Characterization

**Fig. 1:**
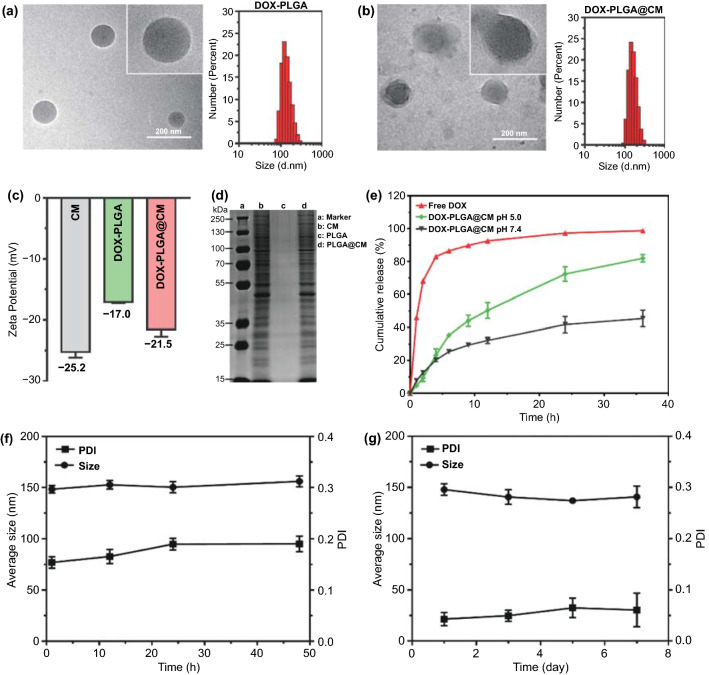
Characterization of DOX-PLGA@CM. TEM and DLS size distribution of DOX loaded PLGA nanoparticles (DOX-PLGA) **a** and MDA-MB-231/Br cell membrane (CM) coated DOX-PLGA nanoparticles (DOX-PLGA@CM) **b**. **c** Zeta potential of CM, DOX-PLGA, and DOX-PLGA@CM. **d** The protein analysis of CM vesicle (lane b), PLGA nanoparticles (lane c), and PLGA@CM (lane d) by SDS-PAGE. Lane a is the protein marker. **e** DOX release profiles from DOX-PLGA@CM. **f** The colloidal stability of DOX-PLGA@CM in PBS containing 10% FBS at 37 °C, and **g** Stability of DOX-PLGA@CM in PBS at 4 °C

The DOX-loaded PLGA nanoparticles (termed as DOX-PLGA) were fabricated according to our previously reported emulsification method [32]. During the preparation, a moderate amount of triethylamine was added to the solvent to increase the hydrophobicity of DOX, which promotes a higher loading efficiency and reduces drug leakage. To extend the half-life of DOX-PLGA in circulation system by minimizing the phagocytosis clear of the reticuloendothelial system (RES) and facilitate the traversing of the BBB and realizing homotypic targeting to BMBC [33], cell membrane (CM) from MDA-MB-231/Br cells with high brain metastatic property was employed to camouflage the surface of the nanoparticle by a co-extrusion method to yield a CM cloaked DOX-PLGA (DOX-PLGA@CM). The hydrodynamic size of DOX-PLGA@CM was 155.6 ± 8.6 nm (Fig. 1b), which was a little larger than its parental DOX-PLGA nanoparticle (146.1 ± 7.9 nm) (Fig. 1a), mainly due to the coating of the CM. Along with the coating, the surface charge of nanoparticles decreased from −17.0 mV (DOX-PLGA) to −22.1 mV (DOX-PLGA@CM), which was close to that of CM formed vesicles (−24.5 mV, Fig. 1c). The morphology of nanoparticles was observed by transmission electron microscope (TEM). DOX-PLGA exhibited a spherical structure, coupled with a bared and smooth surface (Fig. 1a). In contrast, DOX-PLGA@CM showed an apparent core–shell structure, where a shell with a thickness of 8 nm attached to the PLGA core (Fig. 1b), which evidenced the successful fusion of DOX-PLGA with the CM vesicles of BMBC. The final LC and LE were 1.24% and 26.04%, respectively. Sodium dodecyl sulfate polyacrylamide gel electrophoresis (SDS-PAGE) followed by Coomassie brilliant blue staining confirmed that DOX-PLGA@CM has the same set of protein bands as that of pure CM, indicating the retaining of cell membrane proteins after their assembling onto the nanoparticle (Fig. 1d). All the above results validated the successful formation of camouflage nanoparticles with the cell membrane of BMBC.

In contrast to the previously reported DOX@PLGA nanoparticle, which exhibited a biphasic drug release profile [34], DOX release from DOX-PLGA@CM was free of a burst release phase (Fig. 1e). This feature may attribute to the diffusion barrier formed after the coating of the cell membrane. Another possibility is that surface adsorbed DOX and some superficially loaded DOX and may have released from nanoparticles during the coating procedure due to the free diffusion of DOX molecule and mechanical extrusion through the polycarbonate membrane. In addition, it was revealed that DOX release at pH 5.0 (represented lysosomal pH value) was faster than that at pH 7.4 (represented extracellular pH value), suggesting DOX protonation and the rupture of the membrane increased its solubility at the acidic environment. Meanwhile, there was no apparent aggregation, and little change in DLS size of DOX-PLGA@CM over 24 h in PBS supplemented with 10% FBS at 37 °C (Fig. 1f), or within 1 week in PBS at 4 °C (Fig. 1g), indicating its outstanding colloidal stability, which may attribute to a strong repulsion force between the highly negatively charged (−22.1 mV) particles.

### Cellular Uptake of the DOX-PLGA@CM Nanoparticle

**Fig. 2:**
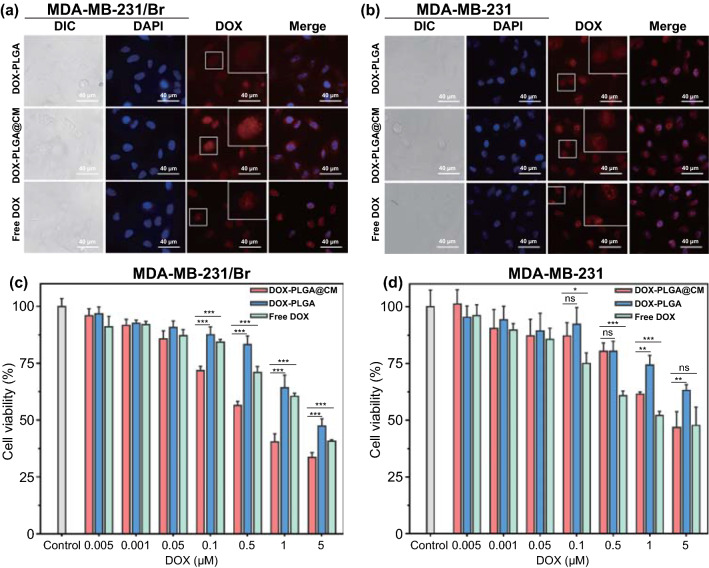
Cellular uptake and cytotoxicity of DOX-PLGA@CM nanoparticle. The cellular uptake of DOX-PLGA, DOX-PLGA@CM and Free DOX in MDA-MB-231/Br cells **a** and MDA-MB-231 cells **b** after 3 h of incubation. Cytotoxicity of DOX-PLGA, DOX-PLGA@CM, and Free DOX for MDA-MB-231/Br cells **c** and MDA-MB-231 cells **d** measured by MTT assay. Data were expressed as mean ± s.d. (*n* = 3). *p < 0.05, **p < 0.01, ***p < 0.001. n.s., not significant

To investigate the homotypic targeting effect of DOX-PLGA@CM, its cellular uptake by MDA-MB-231/Br cells and their parental ones was evaluated with fluorescence microscopy and flow cytometry (FCM). The fluorescence intensity in MDA-MB-231/Br cells treated with DOX-PLGA@CM was significantly higher than that in DOX-PLGA- and free DOX-treated cells (Fig. 2a), and this difference was further confirmed by FCM (Fig. S1). The enhanced internalization effect of DOX-PLGA@CM nanoparticles is attributed to the homotypic adhesive interactions between the membrane proteins of BMBC and its cell source [35]. In contrast, the cellular uptake of DOX-PLGA@CM by parental MDA-MB-231 cells was only slightly enhanced (Figs. 2b and S2), suggesting that the homogenous homing ability of cell membrane camouflage was highly specific due to the differentiated protein expression on cell membrane (Fig. S3), including their abundance and composition. It is noteworthy noting that, besides high retention of DOX in the cell nucleus in nanoparticles treated cells, there was still a significant amount of DOX distributed in the cytoplasm, which could serve as drug depots to continuously supply DOX to the nucleus. Consequently, the DOX concentration in the nucleus could be maintained at a higher level than the cells treated with free DOX.

To verify whether the boosted cellular uptake of DOX-PLGA@CM by MDA-MB-231/Br cells can be translated into an enhanced cell killing effect, we evaluated the cytotoxicity of DOX-PLGA@CM and DOX-PLGA by MTT assay. Figure 2c–d reveals that DOX in all formulations exhibited dose-dependent toxicities to MDA-MB-231 and MDA-MB-231/Br cells. Free DOX and DOX-PLGA exhibited similar cytotoxicity to both cells within the low concentration range (0.005–0.05 µM), while discrepant cytotoxicities were observed at a high dose (0.1 µM). As expected, DOX-PLGA@CM exhibited significantly higher toxicity than DOX-PLGA for both cells in the high dose range. Strikingly, DOX-PLGA@CM killed more MDA-MB-231/Br cells than free DOX in the concentration range from 0.1 to 5 µM, while this phenomenon was not observed in the parental cells, which may attribute to the homotypic targeting effect of CM (Fig. 2a). These discrepant cytotoxicities visually presented from the IC_50_ The IC_50_ of DOX-PLGA@CM for MDA-MB-231/Br cells was 0.289 µΜ, which was significantly lower than that of DOX-PLGA (0.865 µΜ). However, there was nearly no difference in the IC_50_ between the DOX-PLGA@CM (0.805 µΜ) and DOX-PLGA (0.809 µΜ) for MDA-MB-23 cells.

To further probe the potential side effect of DOX-PLGA@CM for normal cells, the cytotoxicity of DOX-PLGA@CM for NIH3T3 cells was investigated. Figure S4 reveals that DOX-PLGA@CM is much less potent in killing NIH3T3 cells than free DOX, suggesting improved therapeutic window for DOX-PLGA@CM in treating BMBC, which was due to the low activity of DOX-PLGA@CM in entering NIH3T3 cells (Fig. S5).

### In Vitro* BBB Penetrating Effect of the DOX-PLGA@CM Nanoparticle*

**Fig. 3:**
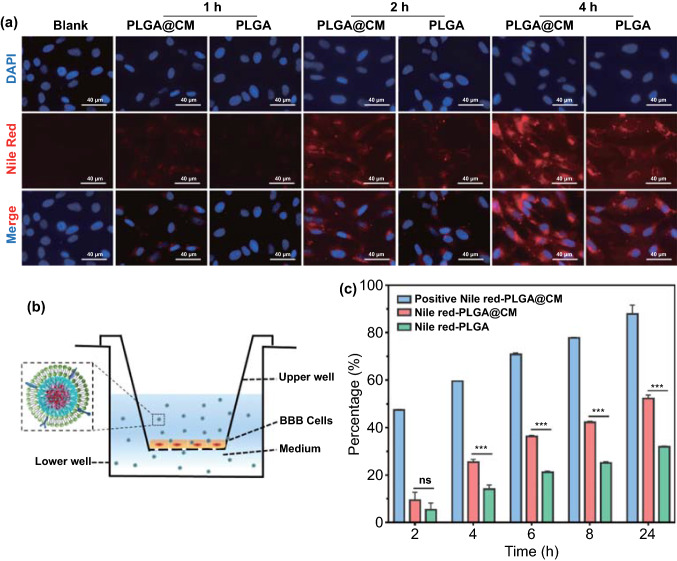
Cellular uptake in hCMEC/D3 cells and in vitro BBB crossing efficiency of Nile red loaded PLGA@CM. **a** Confocal laser scanning microscopy images of hCMEC/D3 cells incubated with PLGA@CM and PLGA nanoparticles for different hours. **b** Schematic of in vitro BBB model. **c** Quantitative analysis of transcytosis efficiency of various nanoparticles through the BBB model in vitro. Data were expressed as mean ± s.d. (*n* = 3). ***p < 0.001. n.s., not significant

To investigate whether the cloak of brain metastatic CM on nanoparticles surface could facilitate nanoparticles transport through the BBB in vitro, we examined the cellular uptake of Nile red loaded PLGA and PLGA@CM by a human brain microvascular endothelial cell (hCEMC/D3), the main component of BBB, by confocal microscopy analysis. As shown in Fig. 3a, the fluorescence signal in hCEMC/D3 increased along with co-incubation time. Moreover, a stronger fluorescence signal was observed in hCEMC/D3 treated with Nile-PLGA@CM than that treated with plain Nile-PLGA nanoparticles at all time points. These results were highly consistent with previous literature report [21], and indicated that the camouflage of brain metastatic cell membrane significantly promoted the internalization of nanoparticles by hCEMC/D3, which was a critical prerequisite for penetrating through the BBB.

Following the cellular uptake, the in vitro BBB penetrating efficiency of nanoparticles was investigated in an in vitro BBB model, as shown in Fig. 3b, where hCEMC/D3 were seeded and cultured on a Transwell insert to form an intact monolayer to mimic brain microvascular endothelial cell layer. In this model, the value of transendothelial electrical resistance (TEER) is an effective indicator to monitor the formation of an endothelial monolayer. When the value of TEER was larger than 200 Ω cm^2^, it meant that the integrity and permeability of the cell monolayer had achieved a similar level as that of in vivo BBB, and therefore could be used for BBB penetration assay [36]. Nile-PLGA and Nile-PLGA@CM nanoparticles were added to the upper chamber, respectively. After being incubated for different time intervals, the penetrated nanoparticles in the lower chamber were collected and quantified by a fluorescence spectrometer. As shown in Fig. 3c, 25.4%, 42.2%, and 52.3% of Nile-PLGA@CM had penetrated the monolayer of hCEMC/D3 cells to the lower chamber at 4, 8, and 24 h, respectively, which were much higher than 14.1%, 25.1%, and 31.9% of Nile-PLGA passed through the BBB at the same time intervals, which indicated that the coating of brain metastatic cells membrane could effectively facilitate nanoparticles across the BBB.

### Pharmacokinetic Properties of the DOX-PLGA@CM Nanoparticle

**Fig. 4:**
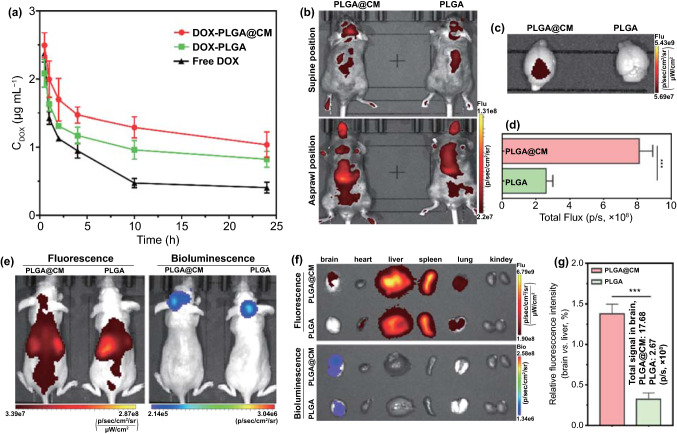
Pharmacokinetics and in vivo distribution of DOX-PLGA@CM. **a** Pharmacokinetics of DOX-PLGA, DOX-PLGA@CM and Free DOX in normal C57BL/6 J mice. **b** Representative in vivo fluorescence imaging of DIR labeled nanoparticles in C57BL/6L mice 3 h after injection. **c** Ex vivo fluorescence imaging of DIR labeled nanoparticles in the brain of C57BL/6 J mice 3 h after injection and its corresponding quantified fluorescence intensity in the brain **d**. **e** In vivo distribution of DOX-PLGA@CM in brain metastases of breast cancer mouse model. Images were taken 3 h after the i.v. injection of DIR labeled nanoparticles. **f** Ex vivo fluorescence images and bioluminescence images for the treatment in (**e**). The quantitative fluorescence intensity in the brain **g** for the treatment in (**f**). Data represent mean ± s.d. for *n* = 3. ***p < 0.001

Theoretical study and research practice have extensively proved that cancer cell membrane camouflaged nanoparticles would inherit most of essential membrane features of their original cells, such as excellent immune escape and prolonged blood circulation by avoiding the phagocytosis and clearance by reticular endothelial system (RES) due to the retained membrane proteins (Fig. 1d), for instance, the “Do not Eat Me” CD47 signal [37]. Therefore, we further studied the pharmacokinetics profiles of DOX-PLGA@CM, DOX-PLGA, and free DOX to verify whether the CM coating can prolong the blood circulation time of DOX-PLGA. As shown in Fig. 4a, free DOX was quickly eliminated from the body, evidenced by the blood elimination half-time (*T*_1/2_) of 3.37 h. The *T*_1/2_ of DOX-PLGA was moderately prolonged to 5.31 h, partially due to the spherical shape and smooth surface of PLGA nanoparticles, which reduced the influence by shearing in the blood [38]. Strikingly, the *T*_1/2_ of DOX-PLGA@CM increased to 8.89 h, significantly longer than those of free DOX and DOX-PLGA. Meanwhile, DOX-PLGA@CM possessed the highest area under the curve (AUC_0-∞_) (94.49 µg L^−1^ h^−1^) compared with free DOX (21.21 µg L^−1^ h^−1^) and DOX-PLGA (48.50 µg L^−1^ h^−1^). These results confirmed that the coating of CM onto the nanoparticle surface could notably prolong the circulation time of the nanoparticles in the blood and thus increased their opportunity in crossing the BBB.

### Biodistribution of the DOX-PLGA@CM Nanoparticle

Encouraged by the excellent performance of CM camouflaged nanoparticles in vitro BBB penetration and in vivo pharmacokinetics, we then estimated whether CM coating could assist the nanoparticles in traversing across the BBB in vivo in healthy C57BJ L^−1^ mice. As shown in the in vivo imaging in Fig. 4b, a strong fluorescence signal was presented in the brain of DIR-PLGA@CM-treated mice 3 h post-injection. In contrast, there was only a weak fluorescence signal in the brain of DIR-PLGA-treated mice at the same time interval. The mice were sacrificed, and ex vivo imaged to quantify the nanoparticle distribution in the brain. Consistent with the in vivo imaging results, the ex vivo fluorescence signal in DIR-PLGA@CM-treated mice brain was much stronger than that of DIR-PLGA-treated mice brain (Fig. 4c), and the difference between them in total flux intensity was around 3.2 folds (Fig. 4d). These results qualitatively and quantitatively evidenced the boosting effect of the membrane of brain metastatic breast cancer cells on propelling nanoparticles across BBB to the brain of healthy mice.

### Establishment of a Breast Cancer Brain Metastases Model

Cancer brain metastasis is an indicator of high malignancy and poor prognosis [6, 39]. For HER2-positive breast cancer patients, more than 30% will progress to brain metastases [6]. Unfortunately, it is still hard to construct a brain metastases model from primary and secondary breast tumors until now. One commonly adopted strategy is direct intracranial implantation of primary cancer cells by stereotactic microinjection to mimic brain metastases and primary glioma. However, this model poorly features the multifocal and infiltrative growth of natural metastasis, especially the BBB integrity is compromised during the operation [40]. Herein, MDA-MB-231 (231/Br), a brain-metastasizing and brain-homing breast cancer cell line derived after two rounds of selection through intracardiac injection and resection from the brain [41], was adopted to construct a breast cancer brain metastases model. 231/Br cells were further engineered to stably express luciferase to yield 231/Br-Luc cells. Figure S6a-b confirms that the luminescence intensity was proportional to the population of the cancer cells. In our study, 231/Br-Luc cells were intracardiac injected into the mice (Fig. S6c) to establish a breast cancer brain metastasis model. Figure S6e-f proves that a brain metastasis tumor model was successfully established 2–3 weeks post-injection despite initially diffused distribution (Fig. S6d). Since the brain tumor colony was formed after 231/Br-luc cells cross the BBB with their unique brain-homing ability, the integrity of the BBB was well-preserved in the model.

### Targeting Effect of the DOX-PLGA@CM Nanoparticle in a Brain Metastatic Tumor Model

To investigate whether the metastatic cancer cell membrane-coated PLGA nanoparticles can traverse the BBB and target a brain metastatic tumor after systemic administration, DOX was replaced with DIR fluorescence probe during the fabrication of DIR-PLGA and DIR-PLGA@CM. Three hours post-injection, IVIS whole body imaging detected strong fluorescence signals in the brain of DIR-PLGA@CM-treated mice (Fig. 4e), mainly located at the bioluminescence signal illumined region. In contrast to others reported PLGA nanoparticle distribution in the brain tumor model established through intracranial implantation, there was nearly no fluorescence signal detected in the brain of DIR-PLGA-treated mice, which validated the integrity of the BBB for our brain metastatic tumor model. To more accurately quantify the distribution of the nanoparticle in different organs, animals were sacrificed to collect the organs for ex vivo imaging. Consistent with their in vivo imaging findings, the fluorescence signals in the isolated brain of DIR-PLGA@CM-treated mice overlapped nicely with that of luminescence signals occupied region (Fig. 4f), suggesting PLGA@CM nanoparticle could effectively cross the BBB and target the metastatic tumor in the brain. On the contrary, only a faint weak fluorescence signal was localized in the brain region of the DIR-PLGA-treated mice, suggesting the commonly accepted enhanced permeability and retention effect (EPR) of tumor is very limited for the brain tumor model established through the intracardiac injection of brain homing cancer cells [21, 42], especially in its early stage of tumor growth when the BBB is intact. There were 3.2 times of difference in total flux intensity between the CM camouflaged nanoparticle and its plain counterpart (Fig. 4g). The results shown in Fig. 4 validated that the coating of metastatic cancer cell membrane could bestow the PLGA nanoparticles with the ability to traverse BBB and target metastatic brain tumor.

### Tumor Growth Inhibitory Effect of the DOX-PLGA@CM Nanoparticle

**Fig. 5:**
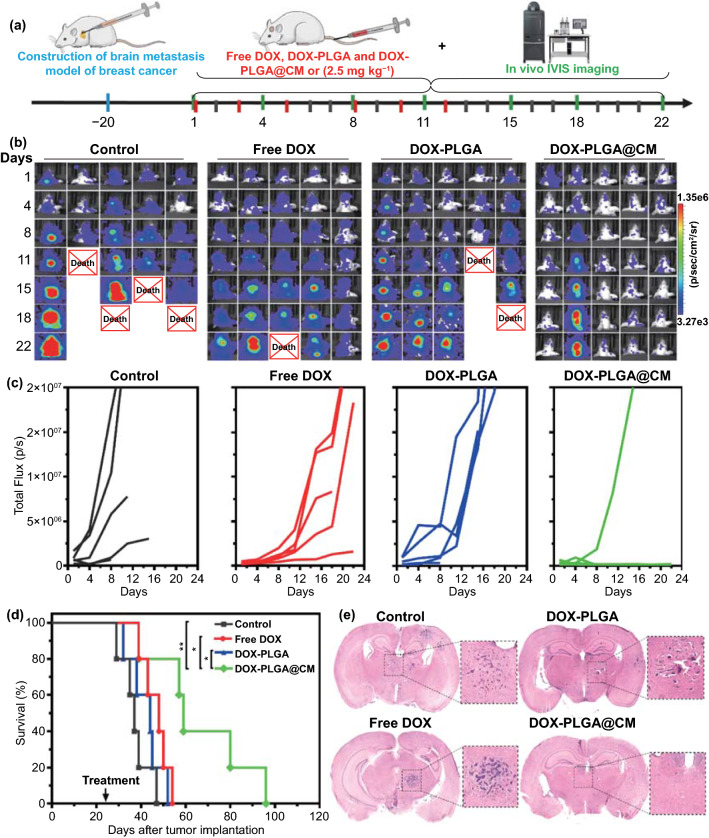
In vivo anticancer efficacy of DOX-PLGA@CM in brain metastases of breast cancer model. **a** The schedule of treatment regimen. **b** In vivo bioluminescence imaging of brain metastases of breast cancer at indicated time. **c** Quantitative bioluminescence intensity of cancer brain metastases corresponding to panel (**b**). **d** Kaplan–Meier survival curves of brain metastases mice received the indicated treatments. *n* = 5. **e** Representative whole brain H&E staining of brain isolated from mice received the indicated treatments. *p < 0.05, **p < 0.01

Cheered by the in vivo prolonged blood circulation time, outstanding performance in traversing the BBB, and homologous homing effect to metastatic brain tumor of PLGA@CM, we further investigated the antitumor efficiency of DOX loaded PLGA@CM (DOX-PLGA@CM) in the above-established breast cancer brain metastases model. The detailed treatment schedule is presented in Fig. 5a. Three weeks post-intracardiac injection of 231/Br-Luc cells, mice developed similarly brain metastatic tumor burdens (based on the luminescence intensity in the brain) were randomly divided into four groups. They received saline, free DOX, DOX-PLGA, and DOX-PLGA@CM treatments via i.v. injection every 2–3 days at the DOX dose of 2.5 mg kg^−1^. Bioluminescence imaging was employed to monitor the progression of the brain metastasis tumor. At the same time, the anticancer effect of DOX-PLGA@CM was measured by quantitatively evaluating the bioluminescence signal intensity in the brain region. As shown in Fig. 5b–c, brain metastases of breast cancer in the control group (saline) grew rapidly. It is worth noting that some mice with a relatively low tumor burden died in their early stage (at 8th day), possibly due to the invasive growth of brain metastases without any intervention and concomitant fatal compression of critical regions in the brain, which further proved the destructiveness and complication of BMBC. Meanwhile, in the free DOX- and DOX-PLGA-treated groups, minor brain metastases growth retardation effects were elicited after the corresponding treatments and some mice dead at the time of lower BMBC growth signal during the treatment similar to the control group, attributing to their rapid blood elimination (Fig. 4a), undesired BBB permeability (Figs. 3 and 4b), and poor distribution in the brain metastases region (Fig. 4). In distinct contrast, except for one animal, there was nearly no bioluminescence signal increase and no mice died in DOX-PLGA@CM-treated mice during the course of treatment, suggesting the super inhibitory effect of DOX-PLGA@CM. In addition, Kaplan–Meier survival analysis further demonstrated that systemic treatment of DOX-PLGA@CM could effectively extend the survival of the mice with brain metastatic tumor. The median survival time for mice received DOX-PLGA@CM treatment was 59 days (Fig. 5d), which was significantly longer than that of saline (37 days), DOX-PLGA (44 days), and free DOX (48 days) treated ones. It was noteworthy that the median survival time for free DOX-treated mice was a little bit longer than that of DOX-PLGA-treated ones, which might be ascribed to the poor distribution of DOX-PLGA in brain metastatic tumor (Fig. 4) due to a limited EPR effect and relatively slow drug release inside cancer cells.

In a separate cohort of mice that received various treatments, the mice were sacrificed on the third day after the last administration (day 15). Their brains and other major organs were collected for histological analysis. H&E staining (Fig. 5e) revealed that numerous metastatic lesions presented throughout the brain in the control group, representing one of the toughest challenges encountered by regular chemotherapy [12]. Similar to that in control, many micro-metastasis lesions were detected in the brain of the mice treated with DOX-PLGA and free DOX. In contrast, only some sporadic micro-metastasis in the brain of mice treated with DOX-PLGA@CM, indicating that DOX-PLGA@CM intervention not only reduced the size of brain metastases but also reduced the number of micro-metastasis lesions, which was consistent with the bioluminescence signal shown in Fig. 5b.

### Systemic Toxicity of DOX-PLGA@CM Nanoparticle

During the course of treatment, there was no significant weight loss among all treatment groups (Fig. S7). The systemic toxicity of the treatments was further investigated through histology assay. Attributed to the relatively low dose of DOX (2.5 mg kg^−1^) given in the treatments, no apparent acute toxic damage was noticed in the major organs, including myocardial injury (Fig. S8), suggesting the excellent biocompatibility and safety of DOX-PLGA@CM for the treatment of breast cancer brain metastasis.

### Discussion

The superiority of DOX-PLGA@CM in fighting against BMBC mainly ascribes to the coating of multifunctional MDA-MB-231/Br cell membrane (CM). Firstly, the camouflage-like clothing of CM bestows DOX-PLGA@CM with prolonged blood-circulation time, (half-lives, Fig. 4a) [43], which would increase the chance of the interplay between DOX-PLGA@CM and BBB. Secondly, the inherited BBB penetrating ability of CM from brain metastatic cells (Fig. 1d) makes DOX-PLGA@CM traversing across the BBB and entering the brain parenchyma easily (Fig. 4b) [21, 26]. Moreover, the general homologous targeting effect of CM [35] makes permeated nanoparticles actively homing to the BMBC (Fig. 4e–f). Consequently, DOX-PLGA@CM effectively inhibited the progression of BMBC. Combined with its wider therapeutic window than free DOX (Figs. 2c and S4), DOX-PLGA@CM could be a safe tool for the treatment of BMBC (Figs. S7 and S8).

## Conclusions

In summary, a Trojan horse approach-based nanocarrier developed by integrating the cell membrane of a brain-homing cancer cell and a PLGA drug depot has been explored for the treatment of brain metastatic breast cancer. With the help of the cell membrane coating, DOX-PLGA@CM nanoparticles were free of burst release, which is a common feature associated with most PLGA nanoparticles. Furthermore, due to the homotypic effect of the cell membrane of MDA-MB-231/Br cells, DOX-PLGA@CM exhibited enhanced cellular uptake and boosted killing potency for MDA-MB-231/Br cells. Functionalized with naturally selected molecules for BBB penetration, DOX-PLGA@CM showed an extended half-life and effectively crossed the BBB in both healthy and early-stage BMBC mouse models. Consequently, DOX-PLGA@CM reached the metastatic tumor lesions in the brain, slowed down cancer progression, reduced tumor burden, and extended the survival time for the BMBC. Benefitting from the easiness of its fabrication and its significant anticancer effect, DOX-PLGA@CM opens a new window for BMBC and other brain metastatic cancers therapy.

## Supplementary Information

Below is the link to the electronic supplementary material.

Supplementary file1 (PDF 815 kb)
