# Supplementary material for: A Therapeutic Sheep in Metastatic Wolf’s Clothing: Trojan Horse Approach for Cancer Brain Metastases Treatment
Source: Nanomicro Lett. 2022 Apr 28;14:114. doi: 10.1007/s40820-022-00861-1 (PMC9050993; doi:10.1007/s40820-022-00861-1)
Supplement: Supplementary file 1 — Supplementary file1 (PDF 815 kb) [file 40820_2022_861_MOESM1_ESM.pdf]

Supporting Information for

A Therapeutic Sheep in Metastatic Wolf's Clothing: Trojan Horse Approach for Cancer Brain Metastases Treatment

Hai-jun Liu[1], Mingming Wang[1], Shanshan Shi[1], Xiangxiang Hu[1], and Peisheng Xu[1,] *

[1]School of Science, Faculty of Science, Engineering and Technology, Swinburne University of Technology, Hawthorn, Victoria 3122, Australia

Department of Discovery and Biomedical Sciences, College of Pharmacy, University of South Carolina, Columbia, SC 29208, United States

*Corresponding author. E-mail: xup@cop.sc.edu (Peisheng Xu)

Supplementary Figures

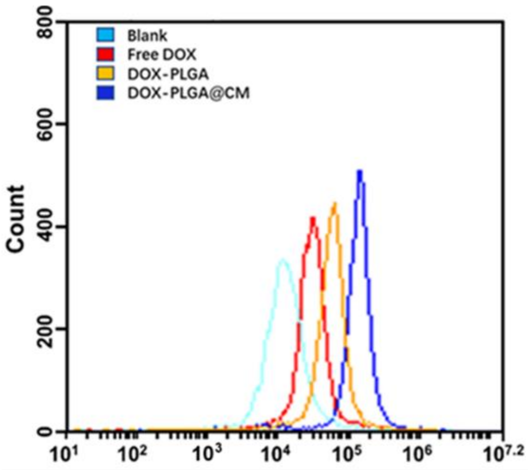

Fig. S1 Flow cytometry spectra of MDA-MB-231/Br after being treated with free DOX, DOX-PLGA, and DOX-PLGA@CM. Cells were analyzed after being incubated with different treatments for 3 h

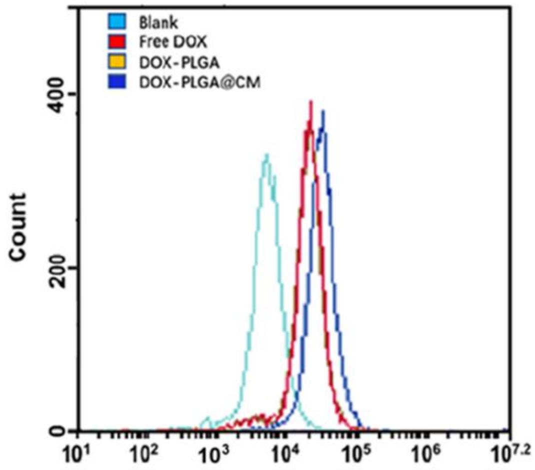

Fig. S2 Flow cytometry spectra of MDA-MB-231 after being treated with free DOX, DOX-PLGA, and DOX-PLGA@CM. Cells were analyzed after being incubated with different treatments for 3

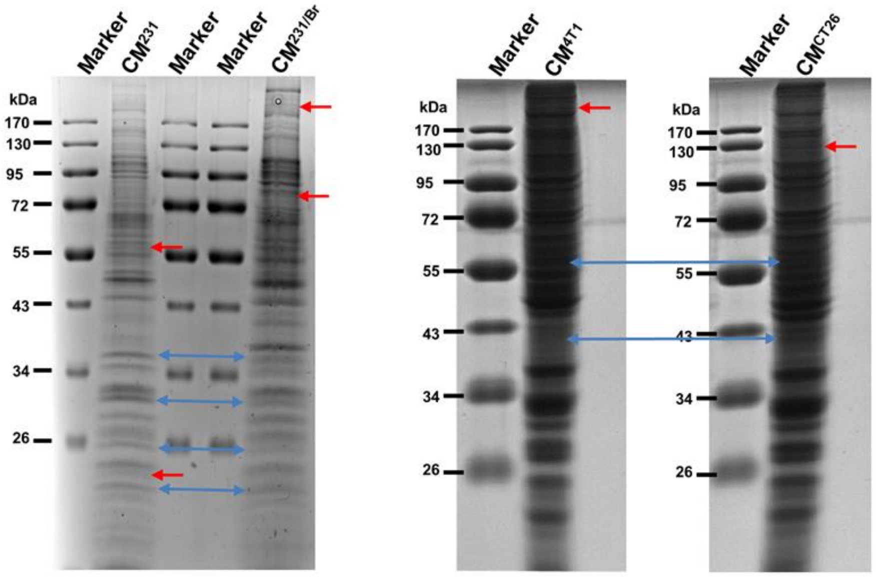

Fig. S3 Characterization of cell membrane proteins from different cell lines by SDS-PAGE. Then red arrow indicates the cell special protein, and blue arrow indicates the common protein but different abundance.

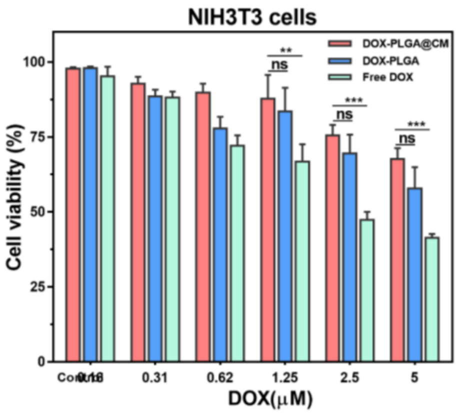

Fig. S4 Cell viability of NIH3T3 cells after the treatments with various concentrations of DOX-PLGA@CM, DOX-PLGA and Free DOX for 24 h measured by the MTT assay

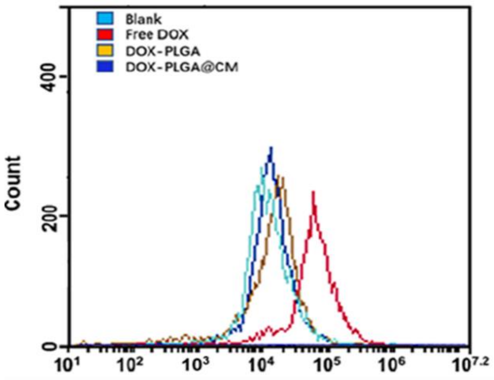

Fig. S5 The flow cytometry spectra of NIH3T3 cells after being treated with free DOX, DOX-PLGA, and DOX-PLGA@CM. Cells were analyzed after being incubated with different treatments for 3 h

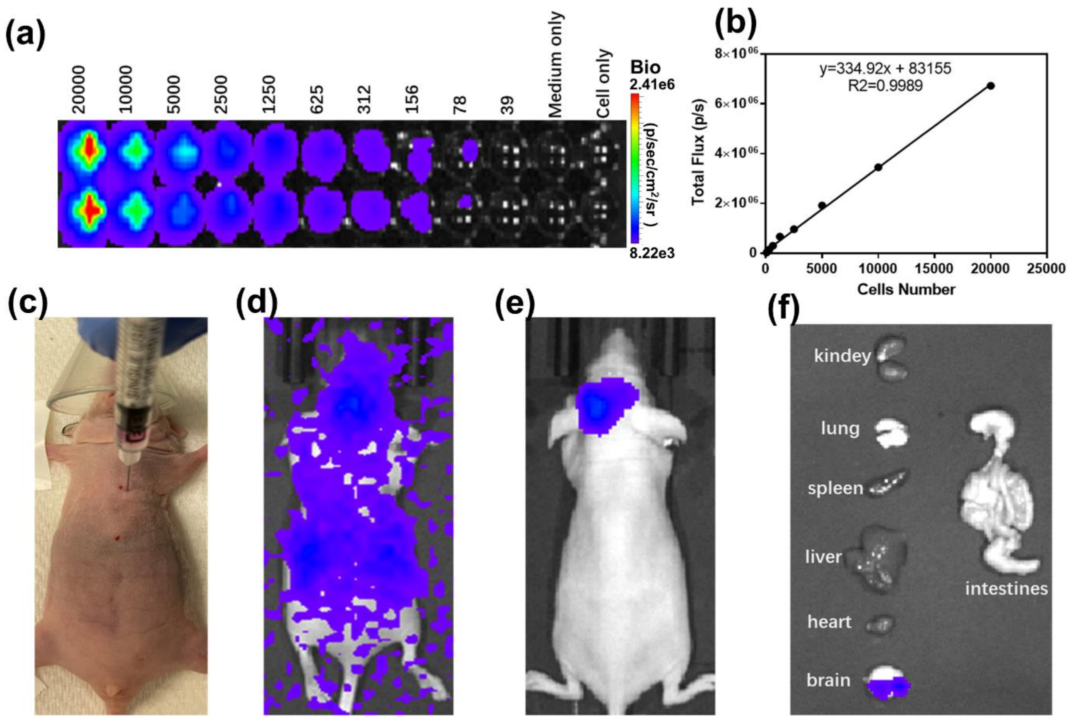

Fig. S6 Establishment of brain metastases of breast cancer model. (**a**) Ex vivo bioluminescence imaging of stably luciferase expressed MDA-MB-231/Br cells. (**b**) Linear correlation between the bioluminescence signal intensity and the number of MDA-MB-231/Br using IVIS Spectrum system. (**c**) Photograph of intracardiac injection of MDA-MB-231/Br to BALB/c nu mouse. In vivo bioluminescence imaging of mice 30 min (**d**) and three weeks (**e**) after the intracardiac injection of MDA-MB-231/Br cells. (**f**) Ex vivo bioluminescence imaging of mice at three weeks after intracardiac injection of MDA-MB-231/Br cells

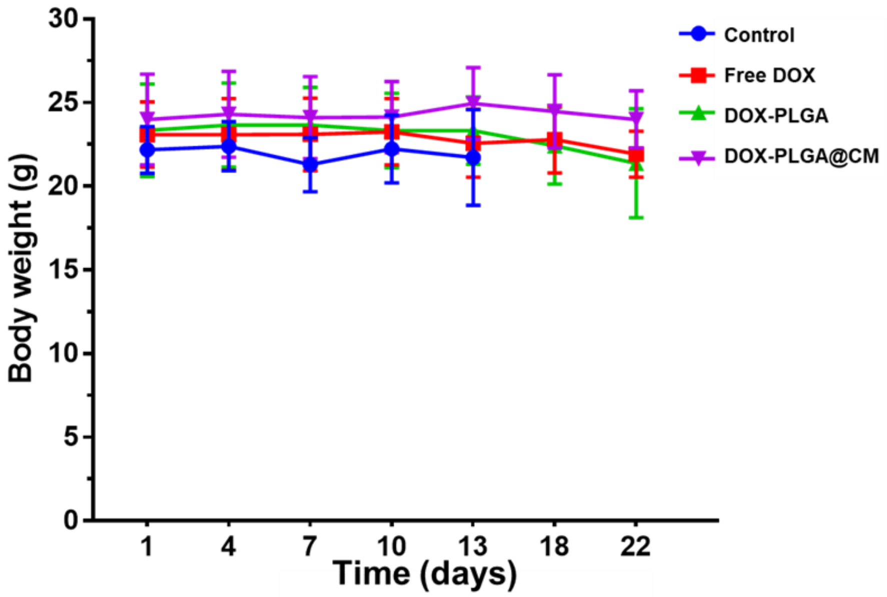

Fig. S7 Body weight change of brain metastases bearing mice in different groups during the treatment

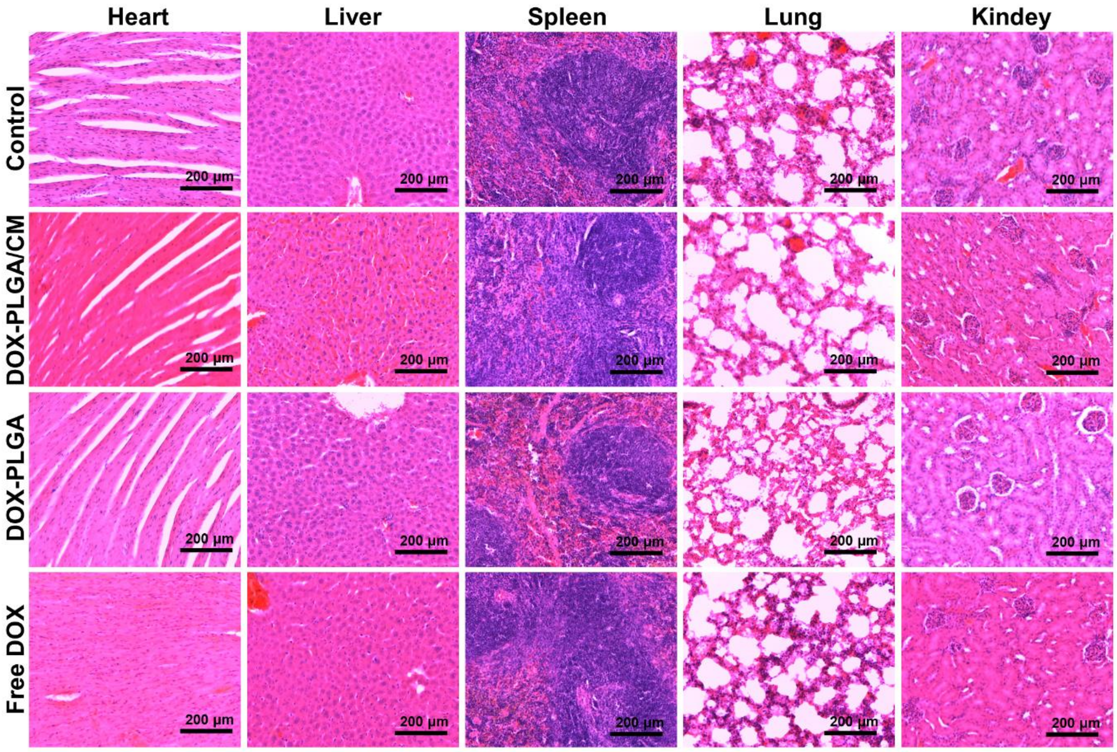

Fig. S8 H&E staining of the major organs (heart, liver, spleen, and kidney) of the mice in different treatment groups
